# Phosphatidylinositol 3-Kinase (PI3K) Orchestrates Aspergillus fumigatus-Induced Eosinophil Activation Independently of Canonical Toll-Like Receptor (TLR)/C-Type-Lectin Receptor (CLR) Signaling

**DOI:** 10.1128/mbio.01239-22

**Published:** 2022-06-13

**Authors:** Axel Dietschmann, Sebastian Schruefer, Stefanie Westermann, Fiona Henkel, Kirstin Castiglione, Ralf Willebrand, Jasmin Adam, Jürgen Ruland, Roland Lang, Donald C. Sheppard, Julia Esser-von-Bieren, Daniel Radtke, Sven Krappmann, David Voehringer

**Affiliations:** a Department of Infection Biology, University Hospital Erlangen and Friedrich-Alexander University Erlangen-Nuremberg (FAU), Erlangen, Germany; b Institute of Clinical Microbiology, Immunology, and Hygiene, University Hospital Erlangen and Friedrich-Alexander University Erlangen-Nuremberg (FAU), Erlangen, Germany; c Center of Allergy and Environment (ZAUM), Technical University of Munich and Helmholtz Center Munich, 80802 Munich, Germany; d Institute of Clinical Chemistry and Pathobiochemistry, School of Medicine, Technical University of Munich, Munich, Germany; e Department of Microbiology and Immunology, McGill University, Montreal, Quebec, Canada; f Department of Medicine, Infectious Diseases, and Immunity in Global Health Program, Centre for Translational Biology, McGill University Health Centre, Montreal, Quebec, Canada; University of Crete; University of Texas Health Science Center

**Keywords:** eosinophils, *Aspergillus fumigatus*, phosphatidylinositol-3-kinase, Toll-like receptor, C-type lectin receptor

## Abstract

Eosinophilia is associated with various persisting inflammatory diseases and often coincides with chronic fungal infections or fungal allergy as in the case of allergic bronchopulmonary aspergillosis (ABPA). Here, we show that intranasal administration of live Aspergillus fumigatus conidia causes fatal lung damage in eosinophilic interleukin-5 (IL-5)-transgenic mice. To further investigate the activation of eosinophils by A. fumigatus, we established a coculture system of mouse bone marrow-derived eosinophils (BMDE) with different A. fumigatus morphotypes and analyzed the secretion of cytokines, chemokines, and eicosanoids. A. fumigatus-stimulated BMDE upregulated expression of CD11b and downregulated CD62L and CCR3. They further secreted several proinflammatory mediators, including IL-4, IL-13, IL-18, macrophage inflammatory protein-1α (MIP-1α)/CC chemokine ligand 3 (CCL3), MIP-1β/CCL4, and thromboxane. This effect required direct interaction and adherence between eosinophils and A. fumigatus, as A. fumigatus culture supernatants or A. fumigatus mutant strains with impaired adhesion elicited a rather poor eosinophil response. Unexpectedly, canonical Toll-like receptor (TLR) or C-type-lectin receptor (CLR) signaling was largely dispensable, as the absence of MYD88, TRIF, or caspase recruitment domain-containing protein 9 (CARD9) resulted in only minor alterations. However, transcriptome analysis indicated a role for the PI3K-AKT-mTOR pathway in A. fumigatus-induced eosinophil activation. Correspondingly, we could show that phosphatidylinositol 3-kinase (PI3K) inhibitors successfully prevent A. fumigatus-induced eosinophil activation. The PI3K pathway in eosinophils may therefore serve as a potential drug target to interfere with undesired eosinophil activation in fungus-elicited eosinophilic disorders.

## INTRODUCTION

Allergic bronchopulmonary aspergillosis (ABPA) is caused by the fungus Aspergillus fumigatus, afflicts about five million patients globally, and is still a noncurable disease (1). ABPA is associated with strong lung eosinophilia. Activated eosinophils may enhance the inflammatory response not only by degranulation and release of toxic proteins but also by secretion of cytokines, chemokines, and lipid mediators. The receptors and signaling pathways involved in activation of eosinophils by A. fumigatus are currently unknown. Studies with macrophages, dendritic cells, and neutrophils identified critical roles of Toll-like receptors (TLRs) and C-type-lectin receptors (CLRs) for recognition of fungal pathogens. Of the TLRs, mainly TLR2, TLR4, and TLR9 have been linked to fungal recognition, but TLR7 has also been implicated in protective antifungal immunity (2, 3). In addition, several CLR family members have been found crucial for the defense of fungi, among them Dectin-1, -2 and -3, Mincle, and more recently, MelLec, which recognize β-glucans, mannose-derived pathogen-associated molecular pattern (PAMPs), and dihydroxynaphthalene-melanin of the fungal cell wall, respectively (4–9). TLRs strongly rely on two downstream signaling adaptors, namely, MYD88, which they share with the interleukin-1 (IL-1) cytokine family receptors and TRIF (*Ticam1* gene) to mount activation of NF-κB, mitogen-activated protein kinase (MAPK), or type I interferon responses (10–14). In contrast, CLRs mainly induce activation of spleen tyrosine kinase (SYK) and protein kinase C-delta (PKCδ) followed by assembly of the caspase recruitment domain-containing protein 9 (CARD9)-B-cell lymphoma 10 (BCL10)-Mucosa-associated lymphoid tissue lymphoma translocation protein 1 (MALT1) (CBM) complex, which then activates the NF-κB cascade, which is crucial for antifungal immunity (15–18).

There is some basic knowledge about TLR and CLR expression in eosinophils, but it remains unclear whether they are required for fungal eosinophil activation (19, 20). It is still unclear whether eosinophils actually express the β-glucan receptor Dectin-1 and can be activated via this route (21–23). Activated eosinophils can secrete large amounts of enzymes and cytokines among other factors and might thereby exacerbate underlying allergic mycoses or hinder fast fungal clearance by phagocytes (24–27). Contrastingly, eosinophils have also been reported to kill allergenic and/or pathogenic fungi such as Alternaria alternata, Pneumocystis murina, and A. fumigatus (22, 28–30). For both scenarios, allergic exacerbation as well as protective immunity, it would be highly desirable to understand in detail the antifungal eosinophil response and its mechanistic regulation. This would certainly help to identify potential drug targets for adequate pharmacological intervention, depending on the particular disease setting.

## RESULTS

### Eosinophils cause fatal lung inflammation in response to intranasal A. fumigatus conidia.

**FIG 1:**
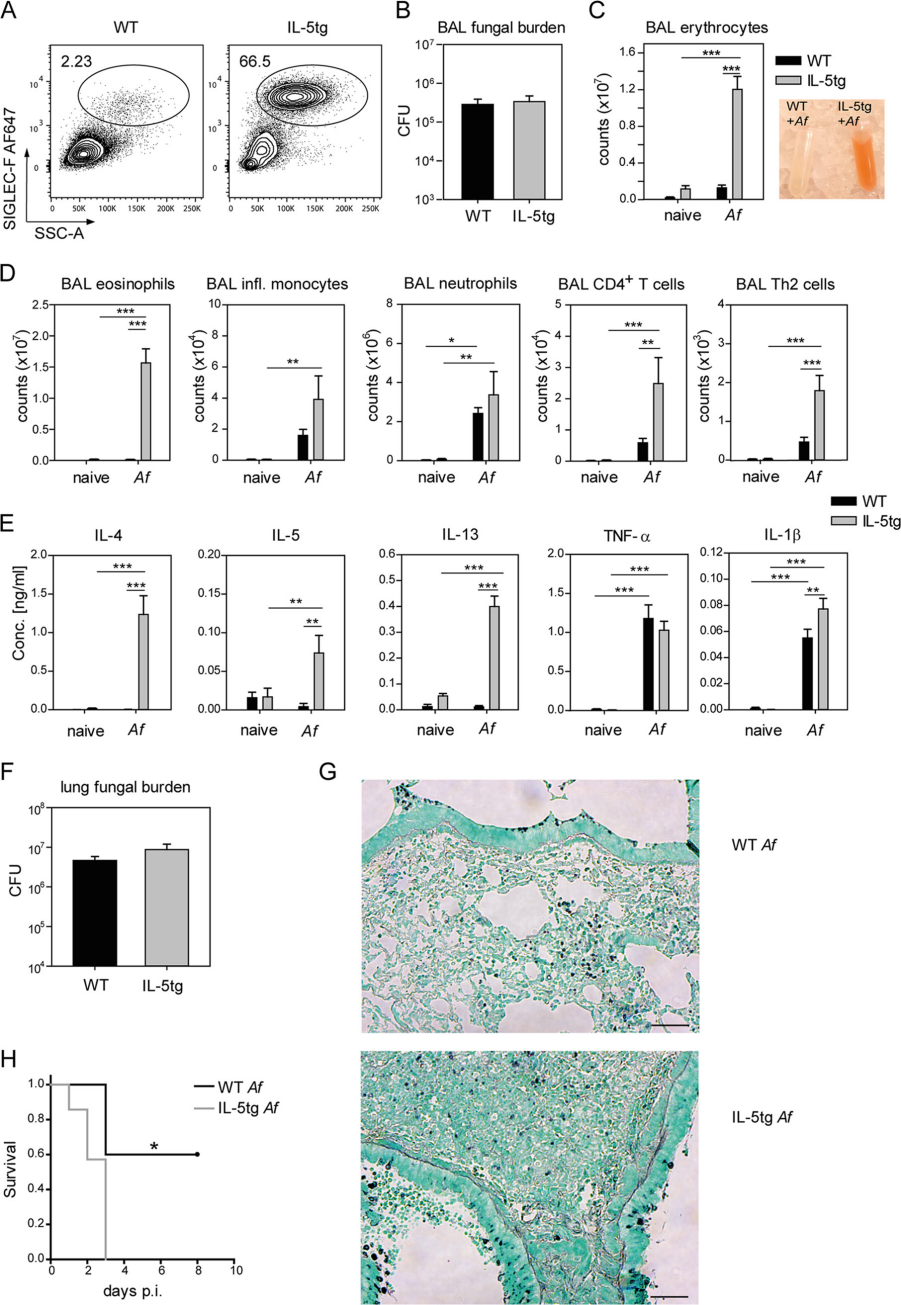
Eosinophil-mediated lung damage in A. fumigatus-infected mice. (A) Representative FACS plots of BAL fluid eosinophils. (B) BAL fluid fungal burden. (C and D) BAL photograph and total number of erythrocytes, eosinophils, inflammatory monocytes, neutrophils, CD4^+^ T cells, and Th2 cells (Th2 defined as 4get^+^) from the BAL fluid of WT or IL-5tg mice crossed to IL-4eGFP reporter (4get) mice 24 h after i.n. infection with 4 × 10^7^ conidia of WT A. fumigatus ATCC 46645. Eosinophils were defined as SIGLEC-F^+^/SSC^high^ from living single cells after excluding SIGLEC-F^high^/CD11c^high^ alveolar macrophages. (E) Cytokines in the BAL fluid. (F) fungal burden of total lung tissue; (G) representative pictures of Grocott-stained lung tissue from WT or IL-5tg mice 24 h after i.n. infection with 4 × 10^7^ conidia of WT A. fumigatus ATCC 46645. The scale bar in the histology pictures equates to 50 μm. (H) Survival analysis of WT and IL-5tg mice after i.n. infection with 10^8^ conidia of the A. fumigatus CEA10 strain. The survival curve shows mice that did not spontaneously die or lost less than 20% of their initial body weight. Displayed data stand for (A and D) 7 to 9, (B and E) 8, (C) 4 to 5, (F and G) 6 to 8 or (H) 5 to 7 mice per group, pooled from (B) 3, (D and E) 4, or (C and F to H) 2 experiments. Bars show the mean + the standard error of the mean (SEM); statistical significance was determined by (B and F) Student’s *t* test, (C to E) two-way ANOVA with the Holm-Sidak *post hoc* test, or (H) log-rank test with *, *P* < 0.05; **, *P* < 0.01; and ***, *P* < 0.001.

Eosinophils can massively accumulate in the lung of ABPA patients and may contribute to lung pathology (31, 32). To investigate whether A. fumigatus-stimulated eosinophils promote tissue damage in the lung, we used a mouse model and administered live A. fumigatus conidia intranasally to wild-type or IL-5-transgenic (tg) mice, which contain abundant eosinophils in most tissues, including the lung (33). One day later, the bronchoalveolar lavage (BAL) fluid and lung parenchyma were analyzed to determine the number of eosinophils and other effector cells, the fungal burden, cytokine concentrations, and the number of erythrocytes as an indicator of lung damage (Fig. 1). Only the BAL fluid of A. fumigatus-challenged IL-5tg mice contained massive numbers of eosinophils and erythrocytes. Inflammatory monocytes, CD4 T cells, and Th2 cells were also increased in the BAL fluid of wild-type mice but even more so in IL-5tg mice. Except for eosinophils, we found no significant differences for the other effector cells in the lung parenchyma (see Fig. S1A and B in the supplemental material). A. fumigatus-challenged wild-type mice showed elevated expression levels of TLR2, TLR6, Dectin-1, and Dectin-2, and a similar trend was apparent in IL-5tg mice, while expression levels of TLR1 and CARD9 did not change (Fig. S1C). The concentrations of IL-4, IL-5, IL-13, and IL-1β upon A. fumigatus challenge were higher in the BAL fluid of IL-5tg compared to wild-type mice, while no difference was observed for tumor necrosis factor alpha (TNF-α) (Fig. 1E). IL-17 and gamma interferon (IFN-γ) were below the limit of detection (data not shown). Although fungal burden and fungal distribution were comparable in both strains of mice (Fig. 1B, F, and G), only IL-5tg mice developed fatal lung damage (Fig. 1H). We therefore investigated the secretion of numerous cytokines, chemokines, and eicosanoids from eosinophils stimulated with different A. fumigatus morphotypes in an *in vitro* coculture system.

10.1128/mbio.01239-22.1 FIG S1 Gating strategy, lung cell infiltrate, and gene expression of WT and IL-5tg mice. Download FIG S1, PDF file, 0.5 MB. Copyright © 2022 Dietschmann et al. 2022 Dietschmann et al. https://creativecommons.org/licenses/by/4.0/ This content is distributed under the terms of the Creative Commons Attribution 4.0 International license.

### Eosinophil activation in an A. fumigatus-eosinophil coculture system.

**FIG 2:**
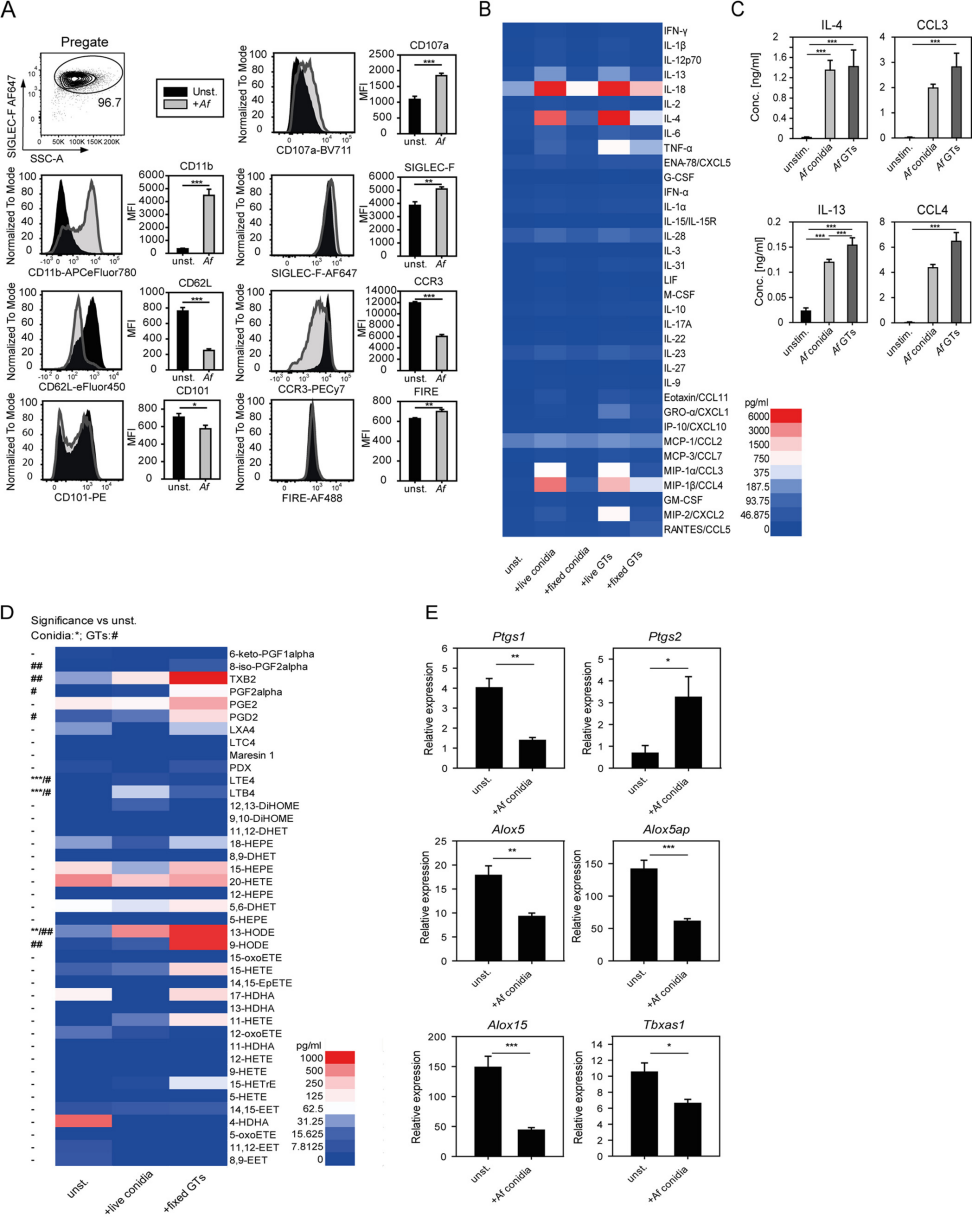
A. fumigatus (*Af*)-induced activation of eosinophils. (A) Flow cytometry staining of a panel of activation-associated surface proteins (activation markers) on BMDE, unstimulated or after 5.5 h of A. fumigatus conidium stimulation. Displayed are representative histograms and the average mean fluorescence intensities (MFI) + SD of three biologically different BMDE cultures measured in one experimental run. Cells were pregated on SIGLEC-F^+^ before retrieving MFI values. Significance was determined by Student’s *t* test; *, *P* < 0.05; **, *P* < 0.01; ***, *P* < 0.001. (B) Result of a cytokine/chemokine multiplex assay screen. Cells were stimulated for 8 h with viable or inactivated A. fumigatus conidia or germ tubes (GT), and the coculture supernatant was analyzed with a bead-based multiplex cytokine assay. Displayed are mean values of technical duplicates of one BMDE culture as a heatmap. (C) Selected confirmatory ELISAs for validation of multiplex assay findings. Displayed are the mean + SD from three biologically different BMDE cultures of one experiment. Significance was determined by one-way ANOVA and Holm-Sidak *post hoc* test or Kruskal-Wallis and Dunn’s *post hoc* test if normality or equal variance was not given. *, *P* < 0.05; **, *P* < 0.01; ***, *P* < 0.001. (D) Heatmap of LC-MS/MS quantified eicosanoid levels in supernatants of BMDE after 8 h of A. fumigatus exposure. The mean of five biologically distinct BMDE cultures per group derived from two experiments is shown. One way-ANOVA with the Holm-Sidak *post hoc* test was performed to determine significant differences from the unstimulated (unst.) control group. If not normally distributed, the Kruskal-Wallis test with the Holm-Sidak *post hoc* or Dunn’s *post hoc* test, if equal variance was not given, were applied. *, significant differences between conidium-stimulated and unst. BMDE; #, between fixed germ tube (GT)-stimulated and unst. BMDE. (E) qRT-PCR expression analysis of indicated eicosanoid synthesis genes from BMDE. Displayed are the means + SEM of unst. and 5.5-h conidium-exposed BMDE of five biologically distinct BMDE cultures of two experiments. Expression levels were normalized to housekeeper *Hprt*. Significance was determined by Student’s *t* test; *, *P* < 0.05; **, *P* < 0.01; ***, *P* < 0.001.

To investigate the A. fumigatus-induced activation of eosinophils *in vitro*, we generated a large homogeneous population of bone marrow-derived eosinophils (BMDE) *in vitro* under defined culture conditions (Fig. S2A). We used flow cytometry to analyze the modulation of surface markers which are known to be regulated upon activation. After coculturing viable A. fumigatus conidia with eosinophils, we observed upregulation of the integrin α_M_ (CD11b), the degranulation marker lysosome-associated membrane protein 1 (LAMP-1) (CD107a), SIGLEC-F, and the F4/80-like receptor FIRE. Downregulation of l-selectin (CD62L), immunoglobulin superfamily member 2 (CD101), and the eotaxin receptor CCR3 (CD193) was observed in parallel (Fig. 2A).

10.1128/mbio.01239-22.2 FIG S2 Gating strategy and inhibitor effect on eosinophil viability. Download FIG S2, PDF file, 0.8 MB. Copyright © 2022 Dietschmann et al. 2022 Dietschmann et al. https://creativecommons.org/licenses/by/4.0/ This content is distributed under the terms of the Creative Commons Attribution 4.0 International license.

We further profiled cytokines and chemokines released from eosinophils after stimulation with different morphotypes of A. fumigatus, namely, viable and fixed conidia and germ tubes (GTs) (Fig. 2B). Conidia contain a cell wall composed of different glycan polymers together with an outer hydrophobin rodlet layer and a melanin layer for protection against environmental hazards. The cell wall of GTs and hyphae has an altered composition of glycan polymers and also contains galactosaminogalactan (GAG) in its outer layer. Therefore, both morphotypes express distinct molecular patterns that could be recognized by cells of the immune system. Viable conidia induced the release of large amounts of IL-4, IL-18, the chemokines macrophage inflammatory protein-1α (MIP-1α)/CC chemokine ligand 3 (CCL3) and MIP-1β/CCL4, and moderate amounts of IL-13. Viable GTs additionally triggered the release of TNF and MIP-2/CXCL2 and moderate amounts of Gro-α/CXCL1. Fixed conidia or GTs showed relatively weaker cytokine and chemokine release from eosinophils. This suggests that metabolic activity of the fungus promotes the activation of eosinophils. We did not detect secreted IL-17A, although it has been reported that eosinophils isolated from the lung of A. fumigatus-challenged mice express IL-17A as revealed by intracellular staining (29, 34, 35). This could mean that eosinophils secrete relatively small amounts of this cytokine or that unknown *in vivo* factors are required for IL-17A expression from eosinophils in this context. Based on our results, we selected four mediators that displayed a pronounced release (IL-4, IL-13, CCL3, and CCL4) and verified their secretion by enzyme-linked immunosorbent assay (ELISA) (Fig. 2C).

Besides cytokines and chemokines, lipid-derived mediators such as eicosanoids also play important roles during inflammation, and eosinophils are a potent cellular source of these factors (36). Thus, we examined the eicosanoid release pattern of A. fumigatus-stimulated eosinophils by mass spectrometry (Fig. 2D). Since A. fumigatus can also produce eicosanoids, we included an inactivated fungus control group to allocate the eicosanoid production to eosinophils and not the fungus (37). Compared to unstimulated eosinophils, GT stimulation promoted the release of the prostaglandins (PGs) D_2_, F_2_α and 8-iso-F_2_α, and thromboxane B_2_, as well as the leukotrienes (LTs) B_4_ and E_4_ from eosinophils. In contrast, conidia triggered only significant production of leukotrienes, though the concentrations were higher than in the GT-stimulated samples.

This indicated that conidial stimulation favored lipoxygenase (LOX) metabolite release, while GT stimulation, rather, promoted the synthesis of cyclooxygenase (COX) metabolites. We furthermore observed high concentrations of the oxidized linoleic acid metabolites 9-HODE (GT stimulation only) and 13-HODE (stimulation with both morphotypes). In addition, we performed reverse transcription-quantitative PCR (qRT-PCR) analysis for the gene transcripts of corresponding eicosanoid synthesis enzymes to investigate if A. fumigatus stimulation would induce their expression in eosinophils. However, we measured a consistent downregulation of all the respective transcripts, with the exception of *Ptgs2* (gene encoding COX-2), which was indeed upregulated in A. fumigatus-stimulated eosinophils (Fig. 2E). Thereby, it became obvious, that eicosanoid release from A. fumigatus-stimulated eosinophils was not necessarily reflected by transcriptional induction of their respective synthesis enzymes.

In summary, A. fumigatus strongly activates eosinophils, which in turn respond with release of proinflammatory cytokines, chemokines, and eicosanoids.

### Canonical TLR and CLR pathways are largely dispensable for eosinophil activation by A. fumigatus.

**FIG 3:**
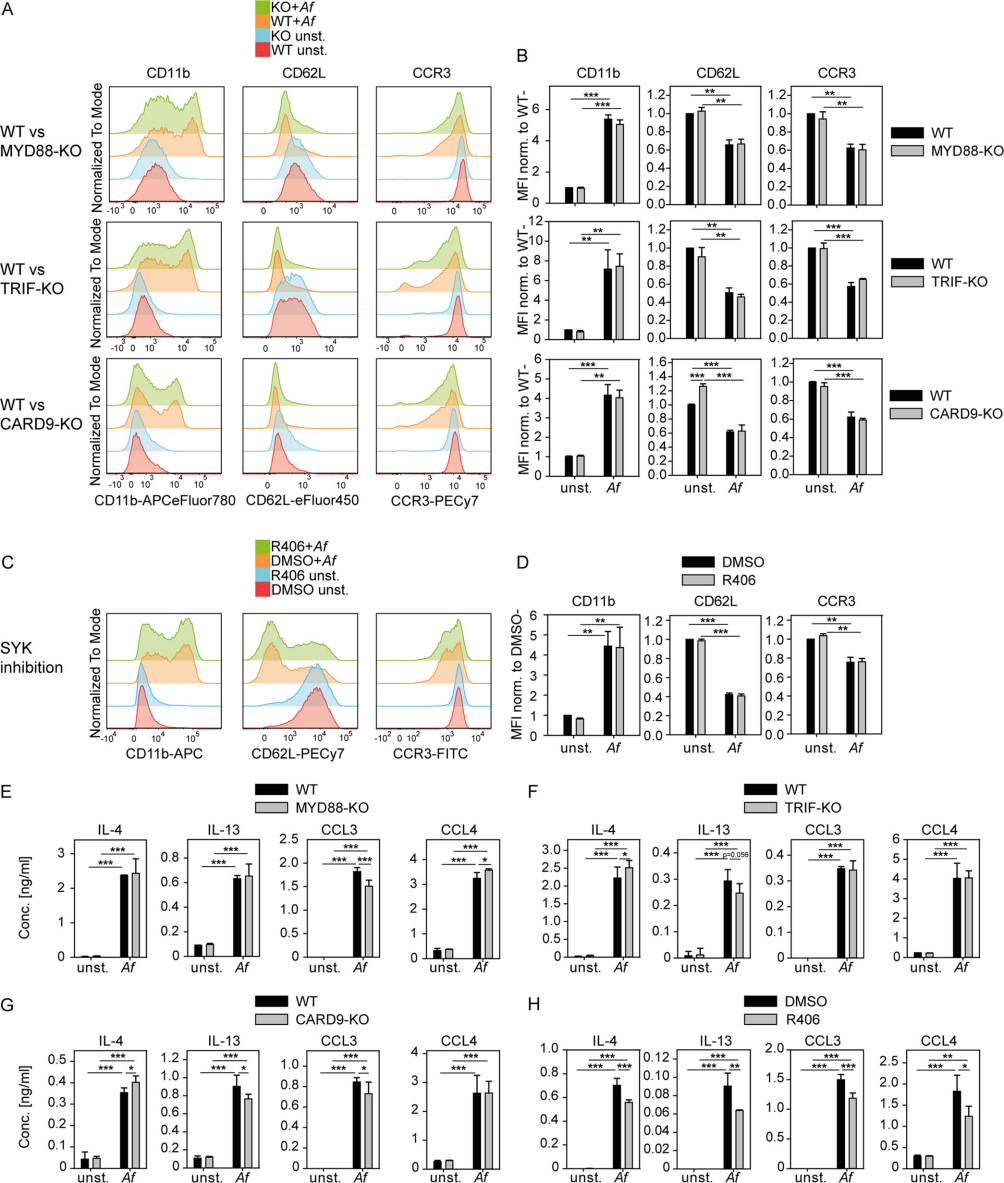
Canonical TLR and CLR signaling is dispensable for A. Fumigatus (*Af*)-triggered eosinophil activation. (A and B) C57BL/6 WT, MYD88-knockout (KO), TRIF-KO, and CARD9-KO BMDE were stimulated with A. fumigatus conidia for 5.5 h, and protein expression of selected activation markers was quantified by flow cytometry. (A) Representative histograms for indicated activation markers and genotypes of unstimulated and A. fumigatus conidium-stimulated eosinophils are shown. (B) Averaged mean fluorescence intensities (MFIs) of the indicated activation marker normalized to the unstimulated WT control of the corresponding experiment are shown. (C and D) WT BALB/c BMDE were pretreated with SYK inhibitor R406 (1 μM) or dimethyl sulfoxide (DMSO) solvent control 30 min before stimulation with A. fumigatus conidia and stained for the same markers as in panels A and B. (C) Representative histograms for indicated activation markers of SYK-inhibited and A. fumigatus conidium-stimulated BMDE, as well as corresponding unstimulated and DMSO controls are shown. (D) Averaged mean fluorescence intensities (MFIs) of the indicated activation marker normalized to the unstimulated DMSO control of the corresponding experiment are shown. (E to H) ELISA for the indicated cytokines in supernatants comparing unstimulated and A. fumigatus conidium-stimulated WT and KO BMDE for (E) MYD88, (F) TRIF, (G) CARD9, or BMDE pretreated with (H) SYK inhibitor R406 (1 μM) or DMSO solvent control. Inhibitor-treated BMDE were analyzed after 5.5 h of stimulation to prevent potential secondary inhibitor-related side effects. Bar graphs show the mean + SEM from pooled data of 2 to 4 biologically distinct eosinophil cultures per group, each implemented as the mean of technical triplicates (A to D) or the mean + SD of technical triplicates of one representative from (E) 3 to 4, (F) 2, (G) 2 to 5, or (H) 3 biologically distinct BMDE cultures per group. Statistical significance was determined by two-way ANOVA with Holm-Sidak *post hoc* testing. *, *P* < 0.05; **, *P* < 0.01; ***, *P* < 0.001.

To further investigate the role of canonical TLR and CLR signaling for A. fumigatus-induced eosinophil activation, we generated BMDE from mice deficient for the TLR adaptors MYD88 or TRIF or from mice lacking the CLR signaling adaptor CARD9 and assessed their capacity to be activated by A. fumigatus. The absence of neither MYD88, TRIF, nor CARD9 caused significant impairment in the upregulation of CD11b or the downregulation of CD62L and CCR3 after stimulation with A. fumigatus compared to the corresponding wild-type (WT) control (Fig. 3A and B). To extend our understanding of the relevance of CLRs for A. fumigatus-mediated eosinophil activation, we additionally included the inhibitor R406, targeting SYK upstream of the CBM complex. However, also, SYK inhibition did not impair the A. fumigatus-triggered activation marker regulation on eosinophils (Fig. 3C and D). Thus, we could not identify a critical role for the central TLR and CLR signaling adaptors for regulation of the activation marker response of A. fumigatus-stimulated eosinophils. Similarly, MYD88, TRIF, and CARD9 were also dispensable for A. fumigatus-induced secretion of IL-4, IL-13, CCL3, and CCL4 from BMDE (Fig. 3E to G). The SYK inhibitor R406 significantly reduced the levels of all four mediators, but the effect was rather modest (Fig. 3H).

We therefore conclude that canonical TLR and CLR signaling is largely dispensable for activation of A. fumigatus-stimulated eosinophils.

### Cell adhesion of A. fumigatus is required for activation of eosinophils.

**FIG 4:**
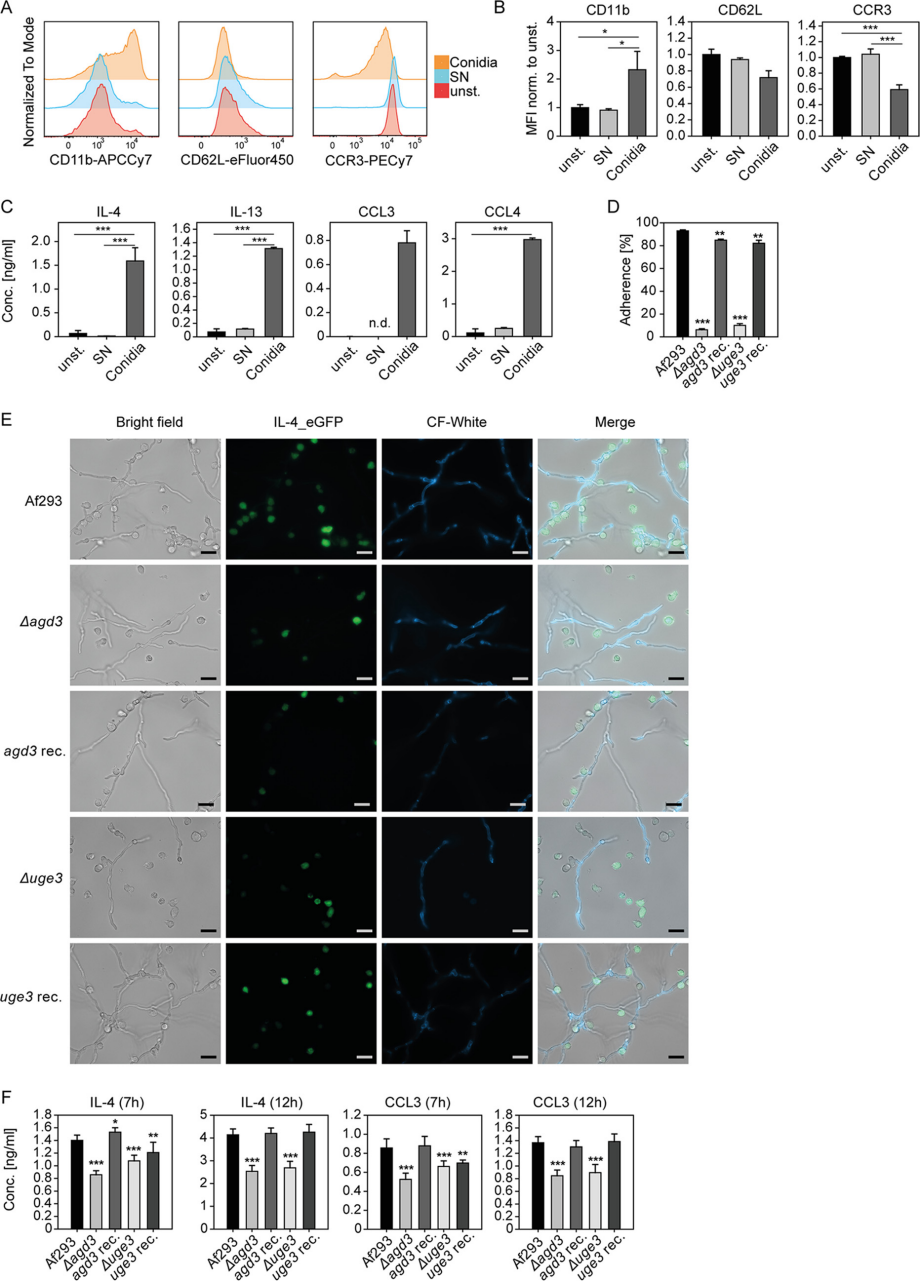
A. fumigatus-triggered activation requires adherence between eosinophils and the fungus. (A to C) C57BL/6 WT BMDE were stimulated with A. fumigatus culture supernatants (SNs) or conidia and subjected to flow cytometry and ELISA for activation measurements. (A) Representative histograms for indicated activation markers of unstimulated, SN-stimulated, or A. fumigatus conidium-stimulated eosinophils are shown. (B) Averaged mean fluorescence intensities (MFIs) of the indicated activation markers normalized to the unstimulated WT control of the corresponding experiment are shown. Data were pooled from 3 biologically distinct cultures of two experiments and are presented as the mean + SEM. (C) ELISAs for the indicated cytokines comparing unstimulated, SN-stimulated, and A. fumigatus conidium-stimulated eosinophils are shown. (D) Quantification of eosinophils attached to hyphae. (E) Exemplary microscopy pictures of BMDE cocultured with calcofluor (CF) white-stained A. fumigatus GAG mutant strains, on which the adherence quantification in panel D was based. Scale bars indicate 20 μm. (F) IL-4 and CCL3 concentrations in supernatants of the indicated cocultures. ELISA and adherence data are displayed as the mean + SD and are representative of 3 biologically distinct cultures per group. Significance was determined by one-way ANOVA and the Holm-Sidak *post hoc* test or Kruskal-Wallis and Dunn’s *post hoc* test, if normality or equal variance was not given. All groups were statistically compared against each other, apart from panels D and F, where all groups were compared to the Af293 A. fumigatus WT strain control group. *, *P* < 0.05; **, *P* < 0.01; ***, *P* < 0.001.

We next investigated whether a soluble, secreted factor from A. fumigatus would be sufficient to trigger the strong activation of eosinophils. Therefore, we compared the activation response of eosinophils exposed to either live conidia or A. fumigatus culture supernatant. None of the activation markers CD11b, CD62L, and CCR3 were significantly regulated by the culture supernatant (Fig. 4A and B). In line with this, the A. fumigatus culture supernatant did not induce cytokine and chemokine secretion from eosinophils (Fig. 4C). As this suggests that physical contact between fungus and eosinophil is indeed necessary for eosinophil activation, we next investigated the activation potential of fungal adherence mutants.

The Δ*agd3* and Δ*uge3* strains both lack functional galactosaminogalactan (GAG), a fungal exopolysaccharide which serves as adhesion and virulence factor. Indeed, both mutants show impaired adherence to cell culture material and to eosinophils which could be restored by gene reconstitution (Fig. 4D and E). Interestingly, we observed significant reductions in the release of IL-4 and CCL3 when eosinophils were stimulated with the A. fumigatus strains lacking *agd3* and *uge3* compared to the nonmutant A. fumigatus strain (Fig. 4F). Reconstitution of these genes restored the eosinophil activation potential of these strains (Fig. 4F). However, neither purified GAG from A. fumigatus cultures (38) or beta-glucan-elicited eosinophil activation (Fig. S3) as deduced from unaltered expression levels of IL-4, IL-13, CCL3, and CCL4. This suggests that eosinophils require direct physical contact to the fungus for being activated, and GAG, beta-glucan, or undefined soluble A. fumigatus-derived factors are not sufficient.

10.1128/mbio.01239-22.3 FIG S3 BMDE are not activated by GAG or beta-glucan. Download FIG S3, PDF file, 0.2 MB. Copyright © 2022 Dietschmann et al. 2022 Dietschmann et al. https://creativecommons.org/licenses/by/4.0/ This content is distributed under the terms of the Creative Commons Attribution 4.0 International license.

### Transcriptional profiling reveals engagement of NF-κB and PI3K-mTOR pathways in A. fumigatus-stimulated eosinophils.

**FIG 5:**
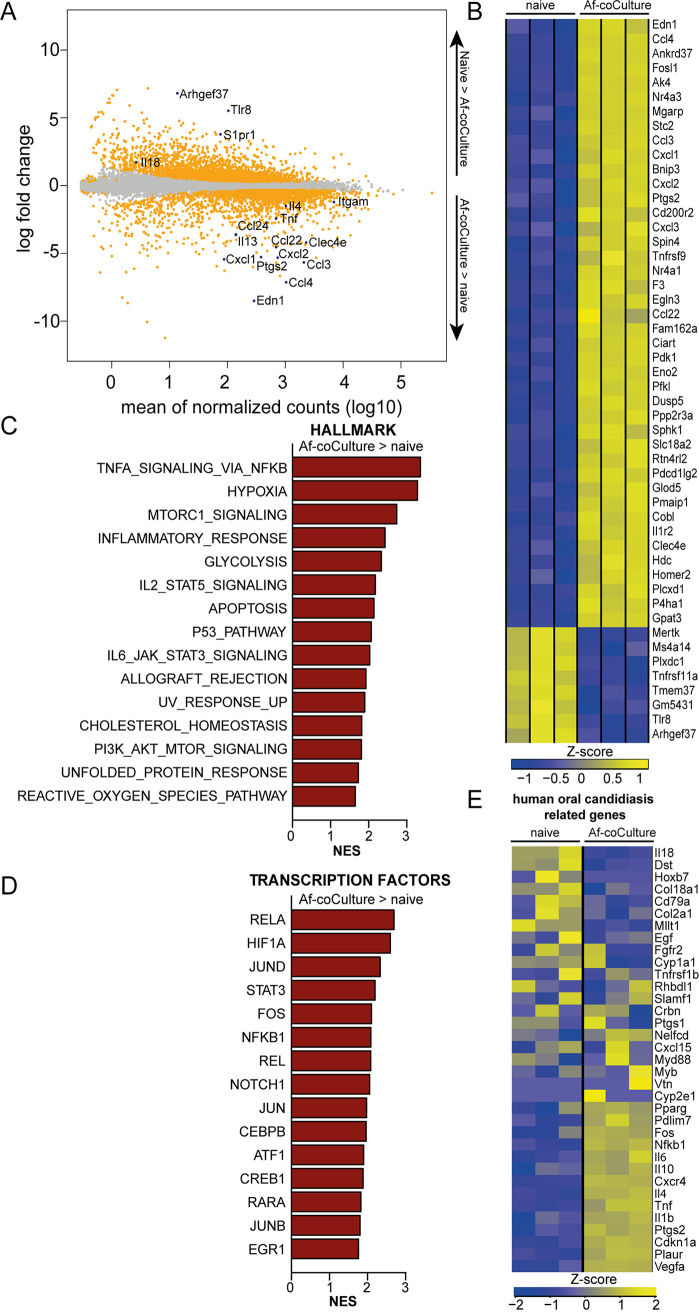
Transcriptional profiling of A. fumigatus-stimulated BMDE reveals enrichment of NF-κB and AKT-mTOR-related pathways. RNAseq and differential expression analysis between eosinophils and A. fumigatus-stimulated eosinophils. (A) MA plot with custom-selected genes highlighted. Significant genes are depicted in orange (adjusted *P* value [*P*_adj_] < 0.05), nonsignificant genes in gray and custom-highlighted in blue. (B) Top 50 differentially expressed genes based on fold change (cutoffs: *P*_adj_ < 0.05; base mean > 100). (C and D) Gene set enrichment analysis of (C) hallmark gene sets or (D) transcription factor gene sets. Shown are the top 15 sets based on normalized enrichment score (NES), all with an FDR of <0.05. (E) Heatmap of murine genes that are homologous for human genes associated with fungus-triggered disease (oral candidiasis gene set of SIDER collection). *n* = 3 biological replicates.

As we did not observe major activation defects by removing central elements of CLR and TLR signaling, and also, no secreted A. fumigatus ligand could be demonstrated to strongly activate eosinophils, we conducted an unbiased transcriptional profiling of A. fumigatus-stimulated BMDE with the goal to identify signaling pathways that could be involved in A. fumigatus-induced eosinophil activation. The 50 strongest induced genes in A. fumigatus-stimulated BMDE included factors that we had already identified by other methods, namely, *Ccl4*, *Ccl3*, *Cxcl1*, *Cxcl2*, and *Ptgs2*, rendering the RNAseq results a credible data set (Fig. 5B). In addition, mRNA expression of *Il13* and *Il4* was also strongly induced (Fig. 5A). We further noticed strong upregulation of the CLR *Clec4e* (Mincle)—a receptor known to be involved in recognition of other fungi, such as *Candida* and *Malassezia*—within the A. fumigatus-stimulated eosinophils (9, 39). However, Mincle-deficient eosinophils were not impaired in their activation marker regulation or cytokine secretion after A. fumigatus stimulation (Fig. S4). We furthermore performed gene set enrichment analysis (GSEA) and among the top 15 hallmark sets found several stress-related sets enriched that were associated with inflammatory response, unfolded protein response, UV/reactive oxygen species (ROS)-related stress responses, and also apoptosis (Fig. 5C). Another interesting aspect was the enrichment of metabolic gene sets, including hypoxia, glycolysis, and cholesterol homeostasis. Enriched cytokine signaling sets comprised IL-2/STAT5, IL-6/STAT3 and, above all, TNF/NF-κB signaling. GSEA further revealed a hallmark gene set related to intracellular mammalian target of rapamycin (mTOR) complex 1 (mTORC1) signaling, as well as another set driven by phosphatidylinositol 3-kinase (PI3K)-AKT-mTOR. The transcription factor database revealed strong enrichment of NF-κB-controlled transcripts, as well as downstream genes of AP-1 factors and hypoxia inducible factor 1α (HIF-1α) (Fig. 5D). To examine if genes differently expressed in A. fumigatus-stimulated eosinophils have potential relevance in the human system, we used human orthologues of our murine data set to perform GSEA against the Side Effect Resource (SIDER) database loaded from GeneSetDB (40, 41). This lists gene names for proteins likely targeted by a drug with a side effect. Gene names associated with the same side effects are then combined to specific gene sets. We found “oral candidiasis” as the top gene set associated with expression changes due to A. fumigatus infection (*P* value < 0.001, false-discovery rate [FDR] < 0.001, normalized enrichment score: −2.23) (Fig. 5E). This highlights that our *in vitro* model resembles a change in genes relevant in fungal-triggered human disease.

10.1128/mbio.01239-22.4 FIG S4 The C-type lectin Mincle is dispensable for the initial activation of BMDE. Download FIG S4, PDF file, 0.3 MB. Copyright © 2022 Dietschmann et al. 2022 Dietschmann et al. https://creativecommons.org/licenses/by/4.0/ This content is distributed under the terms of the Creative Commons Attribution 4.0 International license.

### PI3K inhibition prevents eosinophil activation by A. fumigatus.

**FIG 6:**
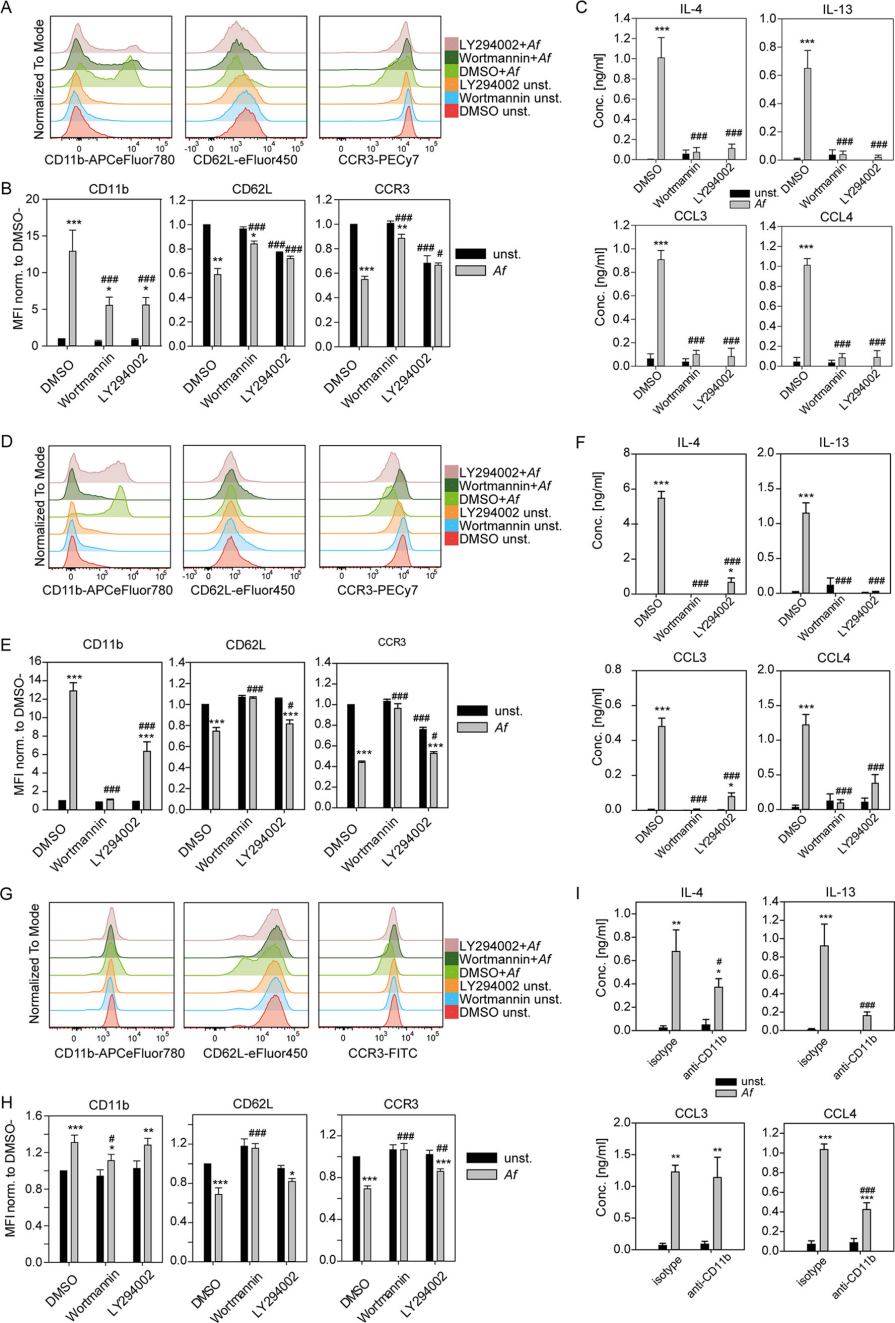
Inhibition of PI3K abrogates A. fumigatus-mediated eosinophil activation. (A to C) WT BALB/c BMDE were pretreated with 0.2 μM wortmannin, 50 μM LY294002, or DMSO solvent control 60 min prior to stimulation with A. fumigatus conidia for 5.5 h and followed by flow cytometry of the cells and ELISA measurements from the coculture supernatant. (A) Representative histograms for indicated activation markers of PI3K-inhibited and A. fumigatus conidium-stimulated eosinophils, as well as corresponding unstimulated and DMSO-controls are shown. (B) Averaged mean fluorescence intensities (MFIs) of the indicated activation marker normalized to the unstimulated DMSO control of the corresponding experiment. Bar graphs show the mean + SEM from pooled data of eight biologically distinct cultures per group from three experiments. (C) ELISA for the indicated cytokines from BMDE coculture supernatants of PI3K-inhibited A. fumigatus conidium-stimulated eosinophils, as well as corresponding uninhibited DMSO-controls are shown. Bar graphs show the mean + SEM of six biologically distinct cultures from two experiments. (D to F) Eosinophils were sorted on day 17 from the BAL fluid of repetitively i.n. A. fumigatus-treated mice, PI3K-inhibited, and A. fumigatus conidium-stimulated *ex vivo* similar to murine BMDE. (D) Representative histograms for indicated activation markers of PI3K-inhibited or DMSO control and unstimulated or A. fumigatus conidium-stimulated eosinophils from flow cytometry analysis are shown. (E) MFIs of the indicated activation marker on *ex vivo* stimulated murine BAL fluid eosinophils normalized to the respective unstimulated DMSO control. (F) ELISA for the indicated cytokines from coculture supernatants of PI3K-inhibited and A. fumigatus conidium-stimulated sorted BAL fluid eosinophils and corresponding DMSO controls are shown. Bar graphs show the mean + SEM of BAL fluid eosinophils sorted from seven mice in two experiments, stimulated individually. (G) Representative histograms for the indicated activation marker on human eosinophils. Human eosinophils were PI3K-inhibited and A. fumigatus conidium-stimulated similar to murine BMDE. (H) MFIs of the indicated activation marker on human eosinophils normalized to the unstimulated DMSO control of the respective donor. Bar graphs show the mean + SEM from pooled data of five biologically distinct human donors per group from three experiments. (I) ELISA for the indicated cytokines from BMDE coculture supernatants of CD11b-blocked (clone 2LPM19c) A. fumigatus conidium-stimulated eosinophils as well as the respective isotype control (clone P3.6.8.2.1) are shown. Bar graphs show the mean + SEM of six biologically distinct cultures from two experiments. Statistical significance was determined by two-way repeated ANOVA with Holm-Sidak *post hoc* testing. *, significant differences between naive and A. fumigatus-stimulated cells in the same treatment group; #, significant differences between the respective treatment group and DMSO control; * or #, *P* < 0.05; ** or ##, *P* < 0.01; *** or ###, *P* < 0.001.

Many of the enriched gene sets (e.g., mTOR, HIF-1α, NF-κB, glycolysis) share a potential upstream signaling regulator: PI3K (42, 43). As we observed in parallel that stimulation with A. fumigatus increased the phosphorylation of AKT in BMDE (Fig. S5), we decided to examine the relevance of the PI3K pathway in eosinophil activation by A. fumigatus. Therefore, we preincubated BMDE with the widely used PI3K inhibitors LY294002 and Wortmannin and then stimulated them with A. fumigatus. Both inhibitors did not significantly affect eosinophil viability (Fig. S2C) but impaired the A. fumigatus-induced change of CD11b, CD62L, and CCR3 expression levels on BMDE (Fig. 6A and B). This effect could be confirmed with primary eosinophils isolated from the lung of mice after repeated administration of 2 × 10^6^ live A. fumigatus conidia (Fig. 6D and E) and with human eosinophils isolated from peripheral blood (Fig. 6G and H and Fig. S2A). In addition, PI3K inhibition caused profound abrogation of the A. fumigatus-induced release of IL-4, IL-13, CCL3, and CCL4 from BMDE and *ex vivo* isolated eosinophils (Fig. 6C and F). These results demonstrate that the PI3K signaling pathway is a critical organizer of eosinophil activation in direct response to the fungal pathogen A. fumigatus.

10.1128/mbio.01239-22.5 FIG S5 A. fumigatus stimulation triggers phosphorylation of AKT in BMDE. Download FIG S5, PDF file, 0.3 MB. Copyright © 2022 Dietschmann et al. 2022 Dietschmann et al. https://creativecommons.org/licenses/by/4.0/ This content is distributed under the terms of the Creative Commons Attribution 4.0 International license.

We further investigated whether CD11b is required for A. fumigatus-induced eosinophil activation, as it has been previously shown that human eosinophils use this receptor for recognition of the fungus Alternaria alternata (22) and that integrins can activate the PI3K pathway (44). Using a blocking antibody against CD11b during A. fumigatus-eosinophil coculture significantly reduced the secretion of IL-4, IL-13, and CCL4, while CCL3 secretion and viability remained unaffected (Fig. 6I and Fig. S2D). This indicates that CD11b is at least one surface molecule on eosinophils involved in A. fumigatus-induced cytokine and chemokine secretion.

## DISCUSSION

We have shown here that eosinophils get activated upon recognition of A. fumigatus and can cause severe pathology in the lung. This result basically confirms a previous report where it has been shown that eosinophils are required for A. fumigatus-induced fatal lung damage (34). However, others have demonstrated that eosinophils can also play a beneficial role and kill the fungus in the lung (29). We observed no reduction in fungal burden in the lung of IL-5tg compared to wild-type mice. This suggests that the detrimental outcome of A. fumigatus-challenged IL-5tg mice is caused by eosinophil-mediated tissue damage rather than invasive aspergillosis. A. fumigatus-activated eosinophils respond with a pronounced release of cytokines, chemokines, and eicosanoids, as well as changes in surface marker expression levels. Among the most prominently upregulated factors were IL-4, IL-13, IL-18, CCL3, CCL4, thromboxane, PGD_2_, and oxidized linoleic acid metabolites, while we observed the strongest A. fumigatus-mediated activation marker regulation for CD11b, CD62L, and CCR3. The activation response required direct contact between fungus and eosinophils, as neither A. fumigatus culture supernatants nor A. fumigatus mutants with impaired attachment were able to induce a considerable amount of eosinophil activation. Thus, in particular, in a scenario where eosinophils come into direct contact with the fungus, their release of cytokines, chemokines, and lipid mediators in addition to toxic proteins stored in intracellular granules promotes tissue damage and exacerbates allergic inflammation (26, 45, 46).

Unexpectedly, eosinophil activation was in major parts not relying on canonical downstream signaling of TLRs or CLRs. This is in considerable contrast to observations of fungal recognition by other immune cell types such as macrophages and dendritic cells (DCs), on which most of our knowledge of fungal recognition is based (16, 47, 48). Transcriptional profiling of A. fumigatus-stimulated eosinophils instead revealed an enrichment of regulated genes related to PI3K-AKT-mTOR signaling. Pharmacological intervention within this pathway by using PI3K small-molecule inhibitors indeed abrogated the majority of A. fumigatus-triggered eosinophil activation patterns. Other publications describe activation of PI3K in pattern recognition receptor (PRR) signaling as well. A. fumigatus has been found to induce phosphorylation of AKT in DCs *in vivo*, but downstream of MYD88 (49). Similarly, the adaptor B-cell adapter for PI3K (BCAP) has been described to translate TLR and IL-1 signals into PI3K activation; however, this also required MYD88 (50, 51). Of note, as an exception to this, one study reported MYD88-independent PI3K activation in IL-1β stimulated neurons, rather fitting to our results (52). PI3K has also been implicated in signaling of CLRs such as Dectins or Mincle or Fc receptors, but downstream of SYK (53–57). Our data with Mincle-deficient eosinophils demonstrate that this receptor is not involved in A. fumigatus recognition by eosinophils, although its mRNA expression is strongly induced. The distinctly superior role of PI3K over SYK in our setup renders the CLR-SYK-PI3K-axis unlikely as the sole activation route and, rather, suggests the involvement of PI3K on more than one level for the entire eosinophil activation process. This includes the PI3K-regulated release of extracellular DNA traps from A. fumigatus-stimulated human eosinophils (58). As we observed regulation of several metabolism genes in our transcriptome data, particularly ones related to glycolysis, it appears plausible that PI3K further promotes a metabolic switch via AKT, mTOR and HIF-1α to provide sufficient energy for the full inflammatory response. In support of this interpretation, it has been shown that fungal melanin sequesters calcium inside phagosomes of macrophages and drives metabolic reprogramming toward glycolysis by an mTOR- and HIF-1a-dependent mechanism (59). This metabolic switch has also been revealed to be involved in the setting of trained immunity and raises metabolic regulation of A. fumigatus-stimulated eosinophils as an interesting aspect for future investigations (42).

PI3K is, furthermore, involved in chemotactic movement and in granulocytes (60–62). As we observed a strong dependency on the adherence between immune cells and fungal elements for eosinophil activation, PI3K signaling might also be required for efficient establishment of cell-cell contact to the fungus. In return, expression of the exopolysaccharide GAG from the fungus appears to be critical for cell adhesion and eosinophil activation. This is an interesting observation, as GAG is commonly seen as an important fungal virulence factor of A. fumigatus (63). Interestingly, GAG has been shown to mask other fungal PAMPs, such as beta-glucans, as A. fumigatus-stimulated DCs were shown to be more activated through Dectin-1, in the absence of GAG (64). It is also required as an adhesin to epithelial cells, very much in line with our adherence data from eosinophil cocultures (64). However, neither GAG nor beta-glucan directly activated eosinophils in our culture system.

The CD11b/CD18 integrin is a likely candidate for an A. fumigatus-activated receptor on eosinophils which may operate to enhance the PI3K-mediated activation. CD11b has been shown on human eosinophils to bind beta-glucans (22) and dimerizes with CD18, which has been described as having signaling capacity via SYK (65, 66). Furthermore, intracellular integrin signaling can also commonly induce the activation of PI3K, closing the circle to the crucial signaling hub molecule for eosinophil activation that we describe here (44). Indeed, we could show here that anti-CD11b blockade significantly reduced the secretion of IL-4, IL-13, and CCL4 from A. fumigatus-stimulated BMDE. PI3K inhibitors have already been experimentally used to counteract symptoms of respiratory allergy, including eosinophilia (67). Our data further indicate that it is possible to interfere with fungus-triggered eosinophil activation using PI3K inhibitors. Future investigations with a focus on A. fumigatus-induced activation of the PI3K pathway in eosinophils could ultimately lead to development of more specific therapeutic approaches for treatment of fungus-induced chronic eosinophil-associated inflammation such as ABPA. Further research is now required to identify the critical ligand-receptor interactions between A. fumigatus and eosinophils from ABPA patients, which would allow for more specific therapeutic interventions. In addition, extending our study to other allergenic environmental fungi could help to identify a common principle of fungal recognition by eosinophils.

## MATERIALS AND METHODS

### Source of bone marrow from different knockout mice.

Mice deficient for *Myd88*, *Ticam1* (TRIF), *Card9*, and *Clec4e* (Mincle) on a C57BL/6 background have been described before and were used as the source for bone marrow (9, 11, 16, 68–70). All mice were kept under specific-pathogen-free (SPF) conditions. Experiments were performed with permission from local authorities at the government of Lower Franconia, Germany.

### Mouse infections.

WT and interleukin-5-transgenic (IL-5tg) (33) mice crossed to IL-4eGFP reporter mice (4get mice) (71) on a BALB/c background were intranasally (i.n.) infected with 4 × 10^7^ ATCC 46645 A. fumigatus conidia to assess fungal burden and numbers of immune cells, erythrocytes, and cytokines in the bronchoalveolar lavage (BAL) fluid 24 h postinfection. BAL fluid was collected by carefully flushing the lungs with 2 × 1 mL of phosphate-buffered saline (PBS) and 0.1 mM EDTA. For analysis of whole lung, lungs were perfused with 8 mL of PBS, and individual lobes were collected separately for flow cytometry, CFU, histology, and RNA. Alternatively, 10^8^ conidia of the virulent A. fumigatus strain CEA10 were applied i.n. to study eosinophil-related mortality. Mice that were suffering from more than 20% body weight loss during these experiments were removed from the study and counted as dead.

### Lung histology.

One lobe of the lung was fixed in 4% paraformaldehyde (PFA) at 4°C overnight. Fixed lungs were dehydrated and embedded in paraffin. Samples were cut with a microtome (Thermo Fisher Scientific) into 2-μm sections, collected onto glass slides and dried overnight at 37°C. Sections were rehydrated in xylol-ethanol row and distilled water. Staining of fungal spores was performed via Grocott’s staining for fungi according to the kit protocol (Morphisto, Offenbach am Main, Germany). After staining, sections were dehydrated with an ethanol-isopropanol-xylol row and embedded in Entellan.

### Generation of bone marrow-derived eosinophils (BMDE).

Mouse bone marrow cells from femurs and tibiae were subjected to red blood cell (RBC) lysis and adjusted to 10^6^/mL in bone marrow medium (BMM) containing 20% fetal calf serum (FCS), 1% nonessential amino acids, 2 mM l-glutamine, 1 mM sodium pyruvate, 100 IU/mL penicillin and 100 μg/mL streptomycin, 19.25 μM 2-mercaptoethanol (all Thermo Fisher Scientific, Waltham, MA) and 25 mM HEPES (Carl Roth, Karlsruhe, Germany) in RPMI 1640 (PAN-Biotech, Aidenbach, Germany). For the first 4 days recombinant mouse stem cell factor (SCF) and FLT3L (PeproTech, Rocky Hill, NJ) were added to the culture (both 100 ng/mL) and afterward replaced by recombinant mouse IL-5 (R&D Systems, Minneapolis, MN) (10 ng/mL) until day 14, with medium including fresh cytokine exchanged by half every 2 days from day 4 on. On day 14 eosinophil culture purity was usually above 90%, assessed by expression of SIGLEC-F and measured via flow cytometry. Bone marrow cells of BALB/c mice were used in all experiments, unless knockout mice with a C57BL/6 background were included in an experimental setup. In this case, accordingly, C57BL/6 WT mice were used as the corresponding control.

### Isolation of human eosinophils.

With approval by the ethics committee of the Faculty of Medicine at the Friedrich-Alexander Universität Erlangen-Nürnberg (FAU) (no. 224_14B) and informed consent, about 50 mL peripheral blood of healthy donors was diluted 1:1 with PBS and subjected to density gradient centrifugation with Biocoll (1.077 g/mL; Bio&SELL) at 1, 000 × *g* for 30 min at room temperature. Supernatant was discarded, and the pellet was subjected twice to red blood cell lysis (15 min and 10 min) in hypotonic lysis buffer (155 mM NH_4_CL, 10 mM KHCO_3_ in H_2_O) on ice. Eosinophils were isolated with the human eosinophil isolation kit (Miltenyi Biotec) according to the manufacturer’s instructions. Purity of the isolated eosinophils (Siglec-8+) was always above 88%.

### Fungal culture.

Aspergillus fumigatus strains were cultured on Aspergillus minimal medium (AMM) (72) for 3 days in the dark at 37°C and afterward washed from the plates with 0.002% Tween 20 in 0.9% NaCl solution (NaCl-Tween) using a swab. Residual hyphal elements were removed by filtration through a 40-μm cell strainer (Corning, Corning, NY). Conidial concentrations were quantified microscopically in a Neubauer improved counting chamber. GTs were generated by growing conidia for 5.5 h in BMM at 37°C and 5% CO_2_. A. fumigatus culture supernatants were generated by 24 h of incubation of 2.5 × 10^6^ conidia/mL in BMM, followed by sterile filtration. In some experiments, conidia and GTs were inactivated by fixation in 4% PFA overnight. The ATCC 46645 WT strain was used in all experiments. As an exception, the Af293 WT was used as a control for the galactosaminogalactan (GAG)-deficient strains Δ*agd3* and Δ*uge3*, which have been generated in this background (64, 73). To measure fungal burden from lungs, tissue was weighed, homogenized with a Bead Ruptor 24 (BioLabProducts, Bebensee, Germany), diluted in PBS, and plated on AMM. CFU were counted after 2 days of incubation at 37°C.

### Coculture of BMDE, lung eosinophils, or human eosinophils and A. fumigatus.

Cocultures were set up in the presence of recombinant mouse or human IL-5 (10 ng/mL) in a 96-well format with 2 × 10^5^ eosinophils and 2 × 10^6^ A. fumigatus conidia (multiplicity of infection [MOI], 10) or 2 × 10^5^ GTs (MOI, 1) or 50% A. fumigatus culture supernatant in a total volume of 200 μL per well. The samples for eicosanoid measurements as well as Western blotting were generated in a 24-well format with 10^6^ BMDE per well and mL and the same MOIs of A. fumigatus. The same MOIs were also used with inactivated fungus. Stimulation was performed at 37°C and 5% CO_2_ in a cell culture incubator. When small-molecule inhibitors were used, cells were preincubated with these before exposure to the fungus. The SYK inhibitor R406 (InvivoGen, San Diego, CA) was used as previously published by 30-min preincubation at 1 μM (74). The PI3K inhibitors wortmannin (Merck) and LY294002 (Cell Signaling Technology, Danvers, MA) were applied following the manufacturer’s recommendations with 60-min preincubation at 0.2 μM or 50 μM, respectively. For CD11b blocking experiments the CD11b clone M1/70 or 5C6 (isotype antibody: LTF2; all Bio X Cell, Lebanon, NH) or the CD11b clone 2LPM19c (isotype antibody: mouse IgG1 kappa clone P3.6.8.2.1; both Thermo Fisher Scientific) was applied with 30- min preincubation at 10 μg/mL.

### Sorting of BAL fluid eosinophils.

To enrich lung eosinophils, 4get WT mice were i.n. sensitized to A. fumigatus ATCC 46645 as described before (75). BALF fluid was collected on day 17, and eosinophils were sorted as 4get^+^ SSC^high^, retrieved in IL-5-supplemented BMM, and seeded at a density of 2 × 10^5^ cells per 96-well plate. Cells were rested for 2 h at 37°C and 5% CO_2_ before proceeding with PI3K inhibition and A. fumigatus stimulation, as described in “Coculture of BMDE, Lung Eosinophils, or Human Eosinophils and A. fumigatus,” above. Eosinophil purity of the sorted cells was above 95%.

### Flow cytometry.

Flow cytometric analyses were performed in accordance with established guidelines (76). Fluorescently labeled antibodies against the following activation-associated surface proteins were used for BMDE: CD11b-APC-eFluor780 (M1/70), CD11c-PerCP-Cy5.5 (N418), CD29-biotin (eBioHMb1-1), CD62L-eFluor450 or -PECy7 (MEL-14), and CD101-PE (Moushi 101) (all Thermo Fisher Scientific); F4/80-like receptor/FIRE-AlexaFluor488 (6F12) and CD170/SIGLEC-F-AlexaFluor647 (E50-2440) (all BD Biosciences, San Jose, CA); and CD107a-BV711 (1D4B), and CD193/CCR3-PECy7 or -FITC (J073E5) (all BioLegend, San Diego, CA). For human eosinophils, the following antibodies against surface proteins were used: CD11b-APC-eFluor780 (M1/70), CD62L-eFluor450 (DREG-56), and CD193/CCR3-FITC (5E8) (all BioLegend) and SIGLEC-8-PE (FAB7975P) (R&D Systems). Cells from BAL fluid or lung were stained with antibodies against CD11b-APC-eFluor780 (M1/70) and CD11c-PerCP-Cy5.5 (N418) (both Thermo Fisher Scientific); and CD170/SIGLEC-F-BV421 (E50-2440), Ly6G-BUV395 (1A8), CD4-BUV395 (RM4-5), and CD8-BUV737 (53-6.7) (all BD Bioscience), Ly6G-PECy7 (1A8), Ly6C-APC (HK1.4) (both BioLegend). Lungs were processed as previously described before staining (75). Cells were stained for 20 min at 4°C in the dark and washed with fluorescent-activated cell sorter (FACS) buffer. If needed, a second identical staining step with BUV395-labeled streptavidin (BD Biosciences) was performed to visualize binding of biotin-labeled antibodies. Cells were filtered through a 70-μm mesh shortly before measuring the samples on a FACS Canto II or LSRFortessa flow cytometer (both BD Biosciences). FACS data were analyzed in FlowJo 10 (BD Biosciences).

### qRT-PCR.

BMDE were harvested after 5.5 h of coculture with the indicated A. fumigatus morphotypes, lysed in RLT buffer (Qiagen, Hilden, Germany) including 0.1 M dithiothreitol (DTT) and frozen at −80°C overnight. RNA was extracted with the column-based RNeasy kit (Qiagen) according to the manufacturer’s instructions. RNA from lung tissue was isolated with TRISure (Bioline, London, UK). Reverse transcription was performed with the Applied Biosystems high-capacity cDNA reverse transcription kit (Thermo Fisher Scientific). qRT-PCRs were performed with the SYBR Select master mix premix (Thermo Fisher Scientific). Samples were measured on a CFX‐Connect instrument (Bio‐Rad Laboratories, Inc., Hercules, CA) or an Applied Biosystems ViiA 7 real‐time PCR system (Thermo Fisher Scientific). Threshold cycle (*C_T_*) value results were normalized to *Hprt* as a reference gene. Primer sequences and PCR conditions can be obtained from the authors upon request.

### Cytokine measurements.

The screening for released cytokines was performed with a ProcartaPlex mouse cytokine and chemokine panel 1A (36-plex) Luminex assay (Thermo Fisher Scientific) according to the manufacturer’s instructions. Undiluted supernatants of cocultures were used after 8-h stimulation time. Measurements were undertaken on a Bio-Rad Bio-Plex MAGPIX multiplex reader. IL-5 was excluded from analysis, since a large amount of recombinant IL-5 was present during culturing to prevent eosinophil apoptosis over the course of stimulation. Individual cytokine release assays for IL-4, IL-13, CCL3, CCL4, IL-1β, IL-5, IL-17, TNF-αn and IFN-γ were performed by enzyme-linked immunosorbent assay (ELISA). Cell culture supernatants were diluted 1:5 or 1:2 in the case of IL-13 before adding them to the ELISA. Cell culture supernatants from experiments of stimulation with cell wall components were all diluted 1:2. BAL samples were used undiluted for ELISA. IL-4 was assayed by an antibody pair of the clones 11B11 (Bio X Cell, West Lebanon, NH) and biotinylated BVD6‐24G2 (BioLegend). IL-13 release was quantified with the murine IL‐13 standard ABTS ELISA development kit (PeproTech). For CCL3, CCL4, IL-1β, IL-5, IL-17, TNF-α, and IFN-γ the corresponding ELISA DuoSet systems (R&D Systems) were used. Para-nitrophenyl phosphate (pNPP) was utilized as a substrate for the chromogenic reaction catalyzed by an alkaline phosphatase coupled to streptavidin (both Southern Biotech, Birmingham, AL) and measured at a wavelength of 405 nm on the Multiskan FC 3.0 instrument (Thermo Fisher Scientific).

### Transcriptional profiling of BMDE from cocultures with A. fumigatus.

A. fumigatus conidia were pregrown for 5.5 h in RPMI 1640. The medium was removed, and eosinophils were added at a conidium-eosinophil ratio of 2:1, along with 10 ng/mL rmIL-5. After 6 h of cocultivation RNA was isolated with the total RNA isolation kit (Fluka, Buchs, Switzerland). Then, 75 mM DTT was added to the lysis buffer to prevent RNA degradation. Samples were treated with an RNase-free DNase set (Qiagen), and RNA integrity was determined with a Bioanalyzer instrument (Agilent, Santa Clara, CA). The TruSeq stranded mRNA kit (Illumina, San Diego, CA) was used for cDNA library preparation. Libraries were 96-bp single-end sequenced on a HiSeq 2500 platform (Illumina). Reads were filtered for a length of at least 60 bp and mapped onto the mouse reference genome GRCm38 with the STAR aligner 2.6.1c. Data were loaded into R (3.5.3; The R Foundation for Statistical Computing, Vienna, Austria), and undetected genes were removed before downstream analysis. DESeq2 (1.20.0) was used on nonnormalized counts to normalize them and to perform differential expression analysis (77). Shrinkage was performed with the apeglm shrinkage estimator (1.4.2) to remove noise, and values were used for the MA plot (78). Heatmaps of log_2_ normalized counts were drawn with the gplots package. For gene set enrichment, GSEA 3.0 software with normalized count values as input and recommended settings (default values but permutation type set to “gene_set”) was used. For hallmark and transcription factor gene set analysis, the MSigDB database 7.1 was employed using a built-in function of the GSEA software to convert mouse to human gene identifiers (79). GSEA of the SIDER database gene sets was also performed with GSEA 3.,0 and genes of the oral candidiasis gene set considered by GSEA 3.0 were used to draw the corresponding heatmap. Data are available via the GEO database (accession no. GSE165694).

### Eicosanoid measurements.

Supernatants of BMDE were harvested after 8 h of coculturing with A. fumigatus conidia or fixed GTs as a control to verify that released eicosanoids were derived from eosinophils and not the fungus. BMDE were not additionally treated with ionophores. The same volume of −80°C precooled methanol was added to the supernatants, and the samples were immediately frozen at −80°C. Eicosanoid concentrations were measured via liquid chromatography tandem mass spectrometry (LC-MS/MS) as described previously (80). Briefly, automated solid-phase extractions were performed on a Microlab STAR robot (Hamilton, Bonaduz, Switzerland). Prior to extraction, all samples were diluted with H_2_O to an MeOH content of 15%, and 10 μL internal standard solution was added. Samples were extracted using Strata-X 96-well plates (30 mg; Phenomenex, Aschaffenburg, Germany) and eluted with MeOH followed by evaporation to dryness under N_2_ stream and redissolved in 100 μL MeOH/H_2_O (1:1).

Chromatographic separation of oxylipins was achieved with a 1260 series high performance liquid chromatography (HPLC) instrument (Agilent, Waldbronn, Germany) using a Kinetex C_18_ reversed-phase column (2.6 μm, 100 by 2.1 mm) with a SecurityGuard Ultra cartridge C_18_ precolumn (both Phenomenex, Aschaffenburg, Germany). The QTRAP 5500 mass spectrometer (Sciex, Darmstadt, Germany), equipped with a Turbo-V ion source, was operated in negative ionization mode. Samples were injected via an HTC PAL autosampler (CTC Analytics, Zwingen, Switzerland) set to 7.5°C. Identification of metabolites was achieved via retention time and scheduled multiple reaction monitoring (sMRM). Acquisition of LC-MS/MS data was performed using Analyst software 1.6.3, followed by quantification with MultiQuant software 3.0.2 (both Sciex).

### Western blotting.

BMDE were cocultured with A. fumigatus conidia or left unstimulated for 1 h. Cells were lysed with RIPA buffer including cOmplete protease inhibitor and PhosSTOP phosphatase inhibitor (both Roche, Basel, Switzerland). Protein extracts were denatured by boiling in Laemmli buffer for 5 min, run on an SDS-PAGE, and then blotted semidry onto a polyvinylidene difluoride membrane. Membranes were soaked in 3% bovine serum albumin (BSA) in TRIS-buffered saline-Tween buffer (TBS-T) for 1 h at room temperature and then incubated with the primary antibody overnight at 4°C. Antibodies against phosphorylated (D9E) and total AKT (C67E7), as well as β-actin (13E5) (all Cell Signaling Technology), were applied. Horseradish peroxidase (HRP)-linked polyclonal goat anti-rabbit antibody (Rockland Immunochemicals, Limerick, PA) was used as a secondary antibody for 1 h, followed by development in SignalFire Plus ECL reagent (Cell Signaling Technology) and luminescence imaging on a Bio-Rad ChemiDoc imaging system.

### Adherence assay.

BMDE were cocultured with A. fumigatus conidia in medium containing 10 ng/mL rmIL-5 in chamber slides for 12 h with a conidium-eosinophil ratio of 1. Adherence was quantified microscopically by counting eosinophils bound to fungal elements per total number of eosinophils in a high-power field of view (20×) on an Apotom V microscope (Carl Zeiss, Oberkochen, Germany).

### Statistics.

Data were evaluated for statistically significant differences in SigmaPlot 12.3. Student’s *t* test was used when comparing two groups against each other, when normality and equal variance were given. Otherwise, the Mann-Whitney U test was applied. In the setting of comparing multiple groups, one-way analysis of variance (ANOVA) or two-way ANOVA were used, depending on the number of nominal variables included in the setup. If normality or equal variance was not given, one-way ANOVA was replaced by the Kruskal-Wallis test. In general, the Holm-Sidak method was used as a *post hoc* test, but Dunn’s *post hoc* test replaced Kruskal-Wallis tests if equal variance failed. Where appropriate, repeated measurement/paired test variants were applied. Significance levels were defined as *P* values of <0.05, <0.01, and <0.001, indicated by one, two, or three stars, respectively. Applied statistics are indicated in the corresponding figure legends.
