# Supplementary material for: Phosphatidylinositol 3-Kinase (PI3K) Orchestrates Aspergillus fumigatus-Induced Eosinophil Activation Independently of Canonical Toll-Like Receptor (TLR)/C-Type-Lectin Receptor (CLR) Signaling
Source: mBio. 2022 Jun 13;13(4):e01239-22. doi: 10.1128/mbio.01239-22 (PMC9426586; doi:10.1128/mbio.01239-22)
Supplement: FIG S1 [file mbio.01239-22-sf001.pdf]

Figure S1

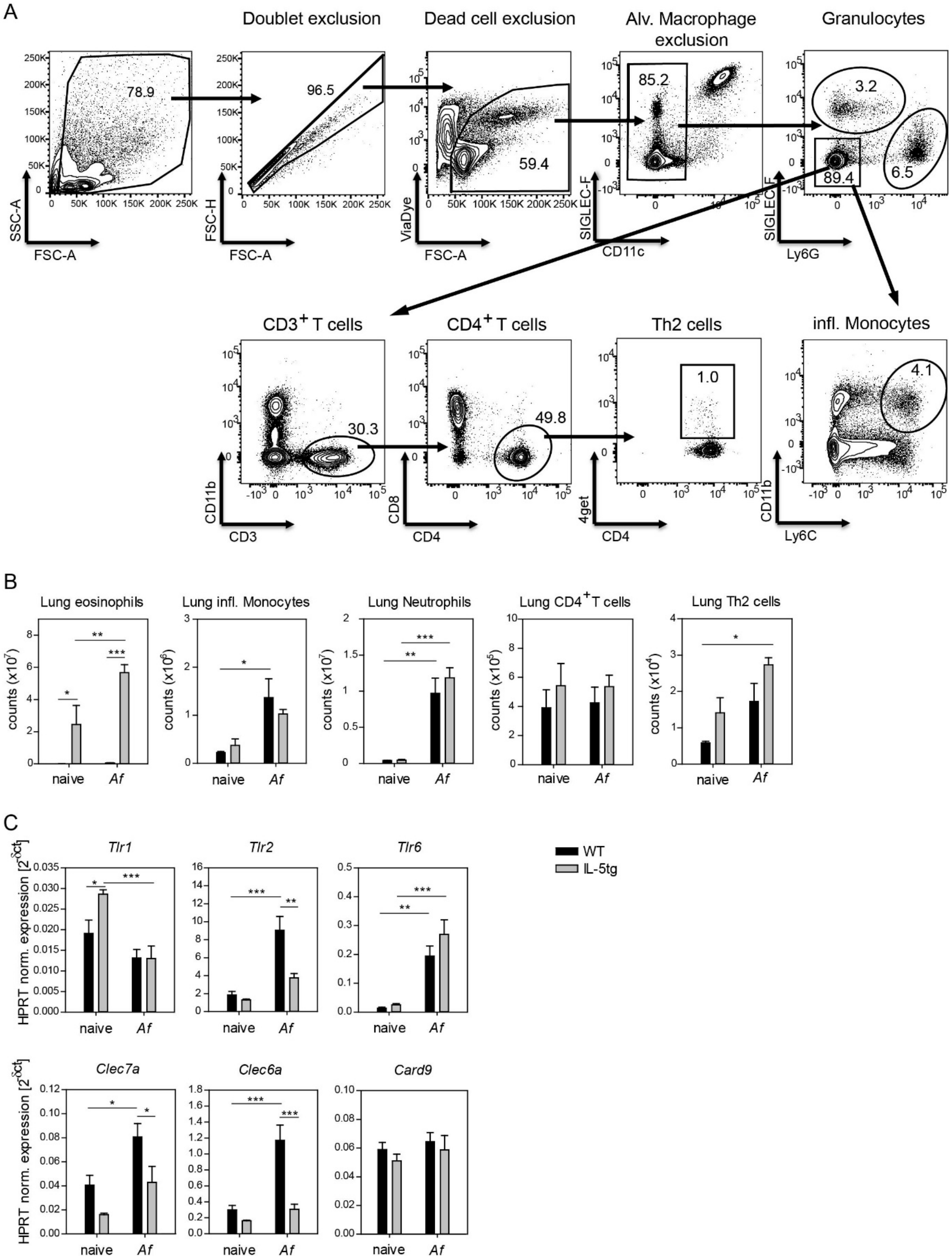
A
Doublet exclusion
Dead cell exclusion
Alv. Macrophage exclusion
Granulocytes
SSC-A
FSC-A
FSC-H
ViaDye
SIGLEC-F
CD11c
Ly6G
78.9
96.5
59.4
85.2
3.2
6.5
89.4
CD3+ T cells
CD4+ T cells
Th2 cells
infl. Monocytes
CD11b
CD3
CD8
CD4
4get
Ly6C
30.3
49.8
1.0
4.1
B
Lung eosinophils
Lung infl. Monocytes
Lung Neutrophils
Lung CD4+T cells
Lung Th2 cells
counts (x10^7)
counts (x10^6)
counts (x10^5)
counts (x10^4)
naive
Af
C
Tlr1
Tlr2
Tlr6
Clec7a
Clec6a
Card9
HPRT norm. expression [2^δct]
WT
IL-5tg

Figure S1. Gating strategy, lung cell infiltrate and gene expression of WT and IL-5tg mice. (**A**) Gating strategy for lung flow cytometry analysis of high dose *Af*-infected WT and IL-5tg mice. Doublet exclusion was followed by dead cell exclusion and exclusion of alveolar macrophages. Out of the SIGLEC-F^+ $CD11c^-$ population, eosinophils were defined as SIGLEC-F^+ $Ly6G^-$ and neutrophils as SIGLEC-F^- $Ly6G^+$. The SIGLEC-F Ly6G double negative population was used as a pre-gate for inflammatory monocytes ($CD11b^+$ $Ly6C^+$) and $CD3^+$ T cells, which were subsequently gated as $CD4^+$ T cells and Th2 cells ($4get^+$). (**B**) Absolute cell counts for total lung eosinophils, inflammatory monocytes, neutrophils, $CD4^+$ T cells and Th2 cells from high dose *Af*-infected WT and IL-5tg mice. Bars display mean count + SEM of three to eight mice per group from two experiments. (**C**) Relative expression of indicated genes in lung tissue of high dose *Af*-infected WT and IL-5tg mice, normalized to *Hprt*. Bars show mean + SEM of four to eight mice per group from two experiments. (**B**, **C**) Statistical significance was determined by two-way ANOVA with Holm-Sidak post-hoc testing. *$p < 0.05$; **$p < 0.01$; ***$p < 0.001$.
