# Supplementary material for: Phosphatidylinositol 3-Kinase (PI3K) Orchestrates Aspergillus fumigatus-Induced Eosinophil Activation Independently of Canonical Toll-Like Receptor (TLR)/C-Type-Lectin Receptor (CLR) Signaling
Source: mBio. 2022 Jun 13;13(4):e01239-22. doi: 10.1128/mbio.01239-22 (PMC9426586; doi:10.1128/mbio.01239-22)
Supplement: FIG S2 [file mbio.01239-22-sf002.pdf]

Figure S2

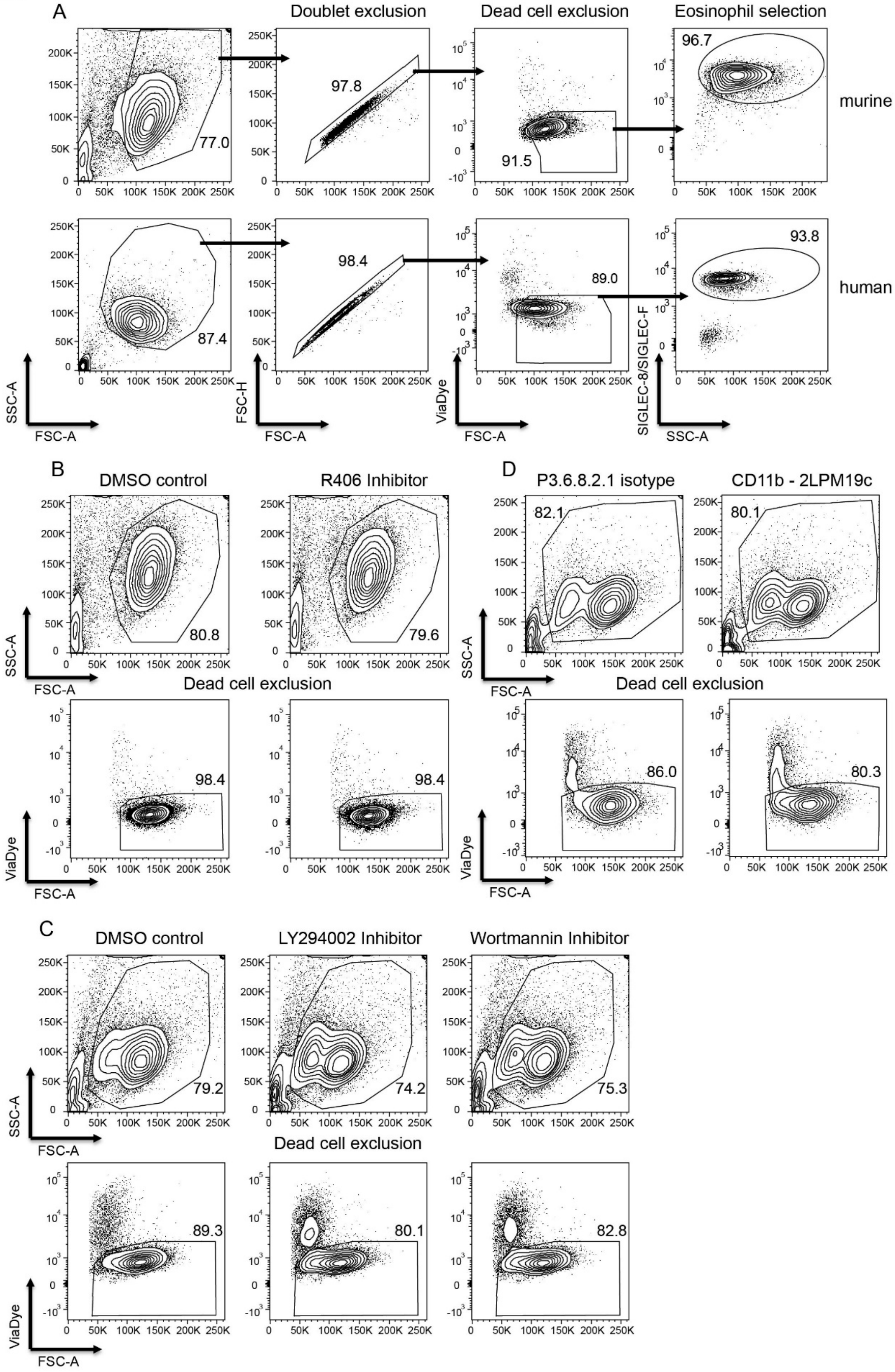
A
Doublet exclusion
Dead cell exclusion
Eosinophil selection
77.0
97.8
91.5
96.7
murine
87.4
98.4
89.0
93.8
human
SSC-A
FSC-A
FSC-H
ViaDye
SIGLEC-8/SIGLEC-F
B
DMSO control
R406 Inhibitor
80.8
79.6
Dead cell exclusion
98.4
98.4
D
P3.6.8.2.1 isotype
CD11b - 2LPM19c
82.1
80.1
Dead cell exclusion
86.0
80.3
C
DMSO control
LY294002 Inhibitor
Wortmannin Inhibitor
79.2
74.2
75.3
Dead cell exclusion
89.3
80.1
82.8

Figure S2. Gating strategy and inhibitor effect on eosinophil viability. (**A**) Eosinophils were gated from live and single cells and defined as SIGLEC-F$^+$ (murine, upper panel) or SIGLEC-8$^+$ (human, lower panel) and SSC^{high}. MFIs of activation markers were derived from this gate. Representative dot plots of FSC/SSC and dead cell exclusion are shown for eosinophils that were treated with (**B**) 1 µM R406 SYK inhibitor or (**C**) 50 µM LY294002 or 0.2 µM Wortmannin PI3K inhibitors or (**D**) 10 µg/mL anti-CD11b antibody clone 2LPM19c.
