# Supplementary material for: Phosphatidylinositol 3-Kinase (PI3K) Orchestrates Aspergillus fumigatus-Induced Eosinophil Activation Independently of Canonical Toll-Like Receptor (TLR)/C-Type-Lectin Receptor (CLR) Signaling
Source: mBio. 2022 Jun 13;13(4):e01239-22. doi: 10.1128/mbio.01239-22 (PMC9426586; doi:10.1128/mbio.01239-22)
Supplement: FIG S3 [file mbio.01239-22-sf003.pdf]

Figure S3

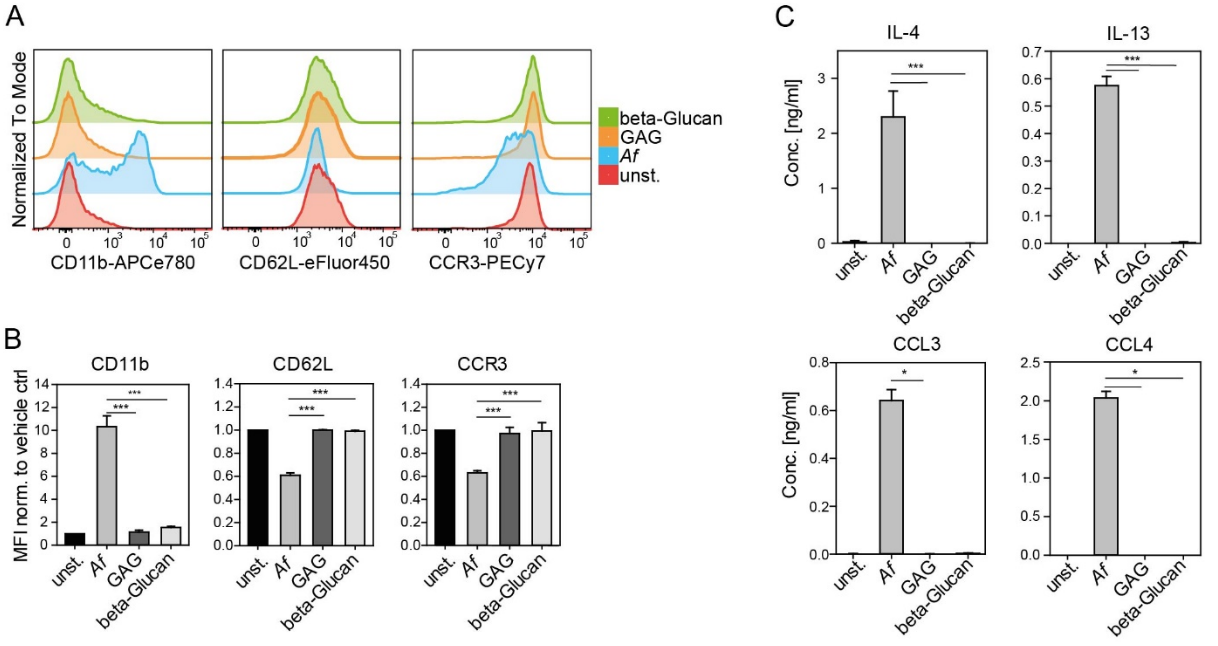

Figure S3. BMDE are not activated by GAG or beta-glucan. BMDE were stimulated with purified GAG or beta-glucan (each 10 µg/ml) or *Af* conidia for 5.5 h followed by flow cytometry analysis and ELISA of culture supernatants. (**A**) Representative histograms of BMDE stimulated with cell wall components or *Af*, as well as unstimulated control. (**B**) Mean fluorescence intensities (MFIs) of indicated activation markers on BMDE normalized to the respective control (HCl diluent for GAG, NaCl diluent for beta-glucan, unstimulated eosinophils for *Af*). (**C**) ELISA for indicated cytokines from BMDE culture supernatants after incubation with cell wall components or *Af* conidia. Statistical significance amongst BMDE stimulated with cell wall components or *Af* was determined by one-way repeated measures ANOVA with Holm-Sidak post-hoc testing (*p <0.05; **p <0.01; ***p <0.001) or , if normality or equal variance failed, by Friedman Repeated measures Analysis on Ranks with Dunn’s post-hoc testing with significance level of *p <0.05.
