# Supplementary material for: Phosphatidylinositol 3-Kinase (PI3K) Orchestrates Aspergillus fumigatus-Induced Eosinophil Activation Independently of Canonical Toll-Like Receptor (TLR)/C-Type-Lectin Receptor (CLR) Signaling
Source: mBio. 2022 Jun 13;13(4):e01239-22. doi: 10.1128/mbio.01239-22 (PMC9426586; doi:10.1128/mbio.01239-22)
Supplement: FIG S4 [file mbio.01239-22-sf004.pdf]

Figure S4

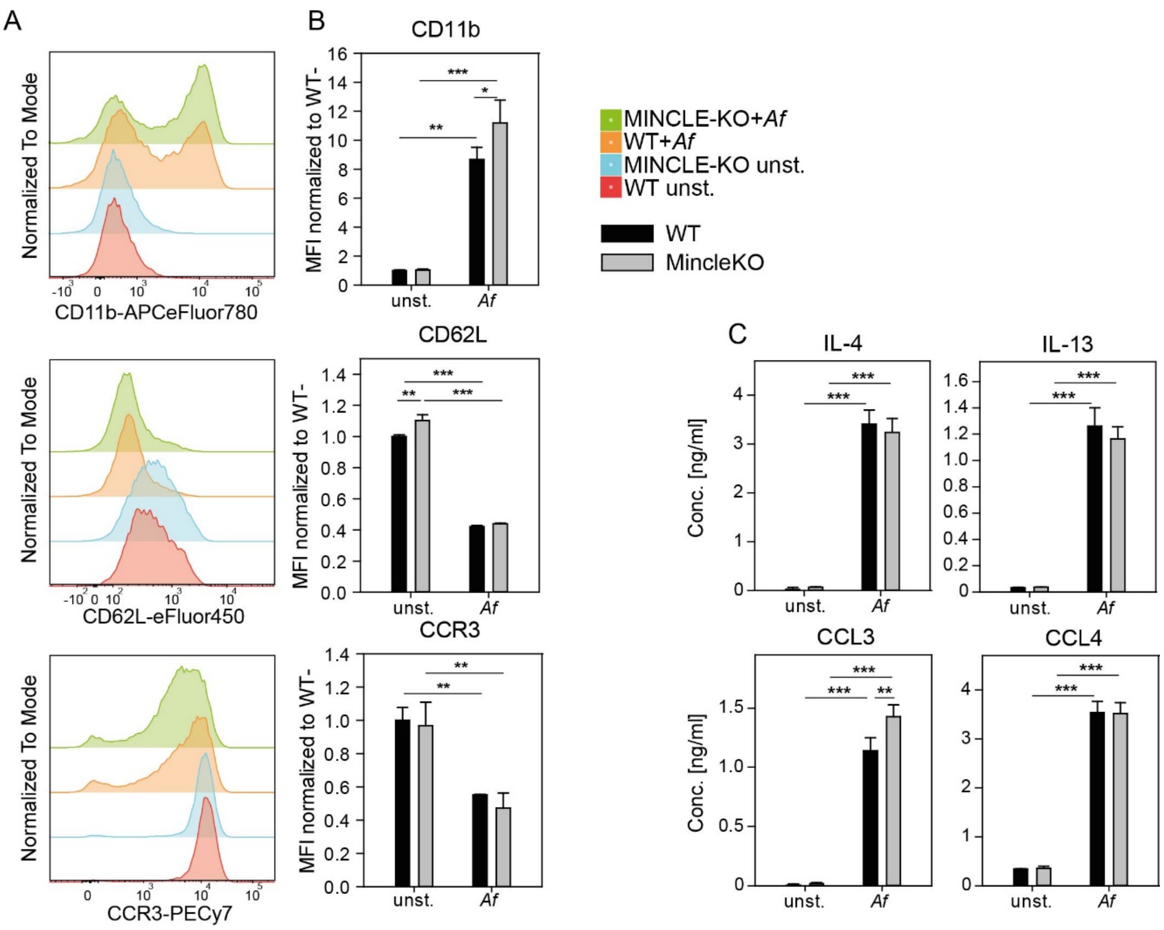

Figure S4. The C-type lectin MINCLE is dispensable for the initial activation of BMDE. C57BL/6 WT and MINCLE-KO BMDE were stimulated with *Af* conidia for 5.5 h and protein expression of selected activation markers was quantified by flow cytometry. **(A)** Representative histograms for indicated activation markers and genotypes of unstimulated and *Af* conidia-stimulated eosinophils are shown. **(B)** Averaged mean fluorescence intensities (MFI)s of the indicated activation marker normalized to the unstimulated WT control of the corresponding experiment are shown. MFI bar graphs show the mean + SEM from pooled data of 2 biologically distinct eosinophil cultures per group, each implemented as mean of technical triplicates. **(C)** BMDE were stimulated with *Af* conidia for 8 h and their supernatants harvested to perform ELISA for the indicated cytokines. ELISA bar graphs show the mean + SD of technical triplicates of one representative from 2 biologically distinct cultures per group. Statistical significance was determined by two-way ANOVA with Holm-Sidak post-hoc testing. *$p < 0.05$; **$p < 0.01$; ***$p < 0.001$.
