# Supplementary material for: Phosphatidylinositol 3-Kinase (PI3K) Orchestrates Aspergillus fumigatus-Induced Eosinophil Activation Independently of Canonical Toll-Like Receptor (TLR)/C-Type-Lectin Receptor (CLR) Signaling
Source: mBio. 2022 Jun 13;13(4):e01239-22. doi: 10.1128/mbio.01239-22 (PMC9426586; doi:10.1128/mbio.01239-22)
Supplement: FIG S5 [file mbio.01239-22-sf005.pdf]

Figure S5

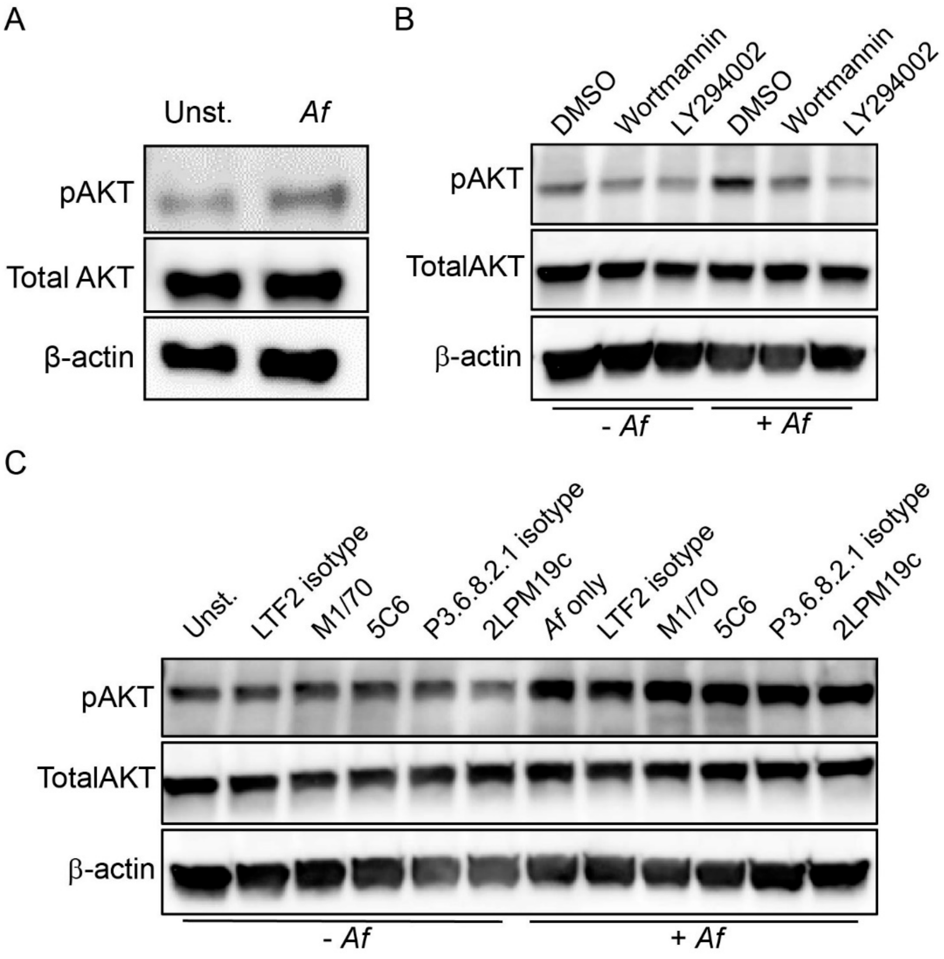

Figure S5. *Af* stimulation triggers phosphorylation of AKT in BMDE. Western blots of phosphorylated and total AKT, as well as β-actin loading control of whole cell lysates of 1 h BMDE-*Af* conidia co-cultures or unstimulated BMDE. (**A**) *Af*-stimulated or unstimulated lysates. (**B**) Whole cell lysates of 1 h co-cultures or unstimulated BMDE after 1 h pre-incubation with 0.2 µM Wortmannin or 50 µM LY294002 PI3K inhibitors. (**C**) Whole cell lysates of 1 h co-cultures or unstimulated BMDE after 30 min pre-incubation with 10 µg/mL anti-CD11b antibody clones M1/70 or 5C6 (LTF2 as corresponding isotype) or 2LPM19c (P3.6.8.2.1 as corresponding isotype).
